# Synthesis, Half-Wave Potentials and Antiproliferative Activity of 1-Aryl-substituted Aminoisoquinolinequinones

**DOI:** 10.3390/molecules19010726

**Published:** 2014-01-08

**Authors:** Juana Andrea Ibacache, Virginia Delgado, Julio Benites, Cristina Theoduloz, Verónica Arancibia, Giulio G. Muccioli, Jaime A. Valderrama

**Affiliations:** 1 Facultad de Química, Pontificia Universidad Católica de Chile, Casilla 306, Santiago 6094411, Chile; 2 Facultad de Química y Biología Universidad de Santiago de Chile, Alameda 3363, Casilla 40, Santiago 9170022, Chile; 3 Facultad de Ciencias de la Salud, Universidad Arturo Prat, Casilla 121, Iquique 1100000, Chile; 4 Instituto de EtnoFarmacología (IDE), Universidad Arturo Prat, Casilla 121, Iquique 1100000, Chile; 5 Facultad de Ciencias de la Salud, Universidad de Talca, Talca 3460000, Chile; 6 Bioanalysis and Pharmacology of Bioactive Lipids Laboratory, Louvain Drug Research Institute, Université Catholique de Louvain, 72 Avenue E. Mounier, BPBL 7201, Brussels 1200, Belgium

**Keywords:** isoquinolinequinones, enaminones, half-wave potential, antiproliferative activity, SAR analysis

## Abstract

The synthesis of a variety of 1-aryl-7-phenylaminoisoquinolinequinones from 1,4-benzoquinone and arylaldehydes *via* the respective 1-arylisoquinolinequinones is reported. The cyclic voltammograms of the new compounds exhibit two one-electron reduction waves to the corresponding radical-anion and dianion and two quasi-reversible oxidation peaks. The half-wave potential values (E^I^_½_) of the members of the series have proven sensitive to the electron-donor effect of the aryl group (phenyl, 2-thienyl, 2-furyl) at the 1-position as well as to the phenylamino groups (anilino, *p*-anisidino) at the 7-position. The antiproliferative activity of the new compounds was evaluated *in vitro* using the MTT colorimetric method against one normal cell line (MRC-5 lung fibroblasts) and two human cancer cell lines: AGS human gastric adenocarcinoma and HL-60 human promyelocytic leukemia cells in 72-h drug exposure assays. Among the series, compounds **5a**, **5b**, **5g**, **5h**, **6a** and **6d** exhibited interesting antiproliferative activities against human gastric adenocarcinoma. The 1-arylisoquinolinequinone **6a** was found to be the most promising active compound against the tested cancer cell lines in terms of IC50 values (1.19; 1.24 µM) and selectivity index (IS: 3.08; 2.96), respect to the anti-cancer agent etoposide used as reference (IS: 0.57; 0.14).

## 1. Introduction

Anticancer quinones are currently the focus of intensive research because of their biological activity and complex modes of action, which differ depending on their particular structure. The biological processes involved with the antitumor activity of quinones are DNA intercalation, bioreductive alkylation of biomolecules, and generation of oxy radicals through redox cycling [1,2,3,4,5]. Aminoquinoline- and aminoisoquinoline-5,8-quinone scaffolds appear as the key structural components of a variety of naturally occurring antibiotics such as streptonigrin [6,7], lavendamycin [8,9], cribrostratin 3 [10], caulibugulones A-C [11], and mansouramycins A-C [12]. This structural array has stimulated the synthesis of novel aminoquinoline- and aminoisoquinoline-5,8-quinones [13,14,15,16,17] mainly directed to extend the spectrum of biological activity on cancer cells. The evidence arising from these studies demonstrate that insertion and change location of the nitrogen substituents at the quinone double bond of the *N*-heterocyclic cores induces significant differences on the half-wave potentials and in the cytotoxic activity. In the search for new aminoisoquinoline-5,8-quinones endowed with anti-proliferative activity we are interested in shedding light on the electronic effects of aryl-substituents bonded at the *N*-heterocyclic ring on the donor-acceptor and the antiproliferative properties of arylaminoisoquinolinequinones. Herein we wish to report the synthesis and half-wave potentials of a series of 1-arylaminoisoquinoline-5,8-quinones, together with their *in vitro* antiproliferative activities on two cancer cell lines.

## 2. Results and Discussion

### 2.1. Chemistry

Isoquinolinequinones **3a**–**d** and **4a**–**d** were selected as precursors of the designed arylaminoisoquinolinequinones. Compound **3a** and **4a** were prepared from 2,5-dihydroxybenzaldehyde (**1a**) and enaminones **2a,b** respectively, by using a previously reported one-pot procedure [16,17,18]. Through the same procedure, compounds **3b**–**d** and **4b**–**d** were accomplished from the corresponding acylhydroquinones **1b**–**d** [19,20] and enaminones (Table 1). 4-Aminopent-3-en-2-one (**2b**) was prepared in 80% from acetyl acetone and ammonium carbamate according to the method reported by Litvié * et al.* [21].

**Figure 1:**
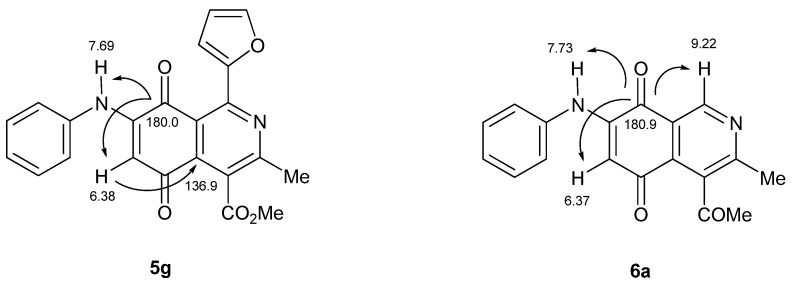
^3^*J*_C,H_ correlations for compounds **5g** and **6a** accomplished by HMBC.

The acid-induced amination of quinones **3b**–**d** and **4a**–**d** with aniline and 4-methoxyaniline was examined. The reactions were conducted at room temperature in ethanol and monitored by TLC. The reactions of quinones **3b**–**d** and **4a**–**d** proceed in high yield and in a regioselective manner to give a sole regioisomer where the nitrogen substituent occupies the 7-position (Table 2). The structures of the new compounds were established on the basis of their nuclear magnetic resonance (^1^H-NMR, ^13^C-NMR, 2D-NMR) and high resolution mass spectra (HRMS). For instance, the position of the phenylamino group at C-7 in **5g** was unequivocally settled from ^3^*J*_C,H_ couplings between the carbon at C-8 with the amino proton and with the proton at C-6, which is coupled with the carbon at C-4a (136.9 ppm). In the case of aminoquinone **6a**, the position of the phenylamino group was assigned on the basis of the ^3^*J*_C,H_ coupling between the carbon C-8 with the proton at C-6, the proton at the C-1 and the amino proton (Figure 1).

**Table 1:** Preparation of 1-arylisoquinoline-5,8-quinones **3** and **4**. 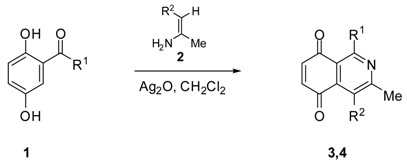

Precursors		Product	R^1^	R^2^	Yield (%) ^a^
**1a**	**2a**	**3a**	H	CO_2_Me	86 ^b^
**1b**	**2a**	**3b**	phenyl	CO_2_Me	60
**1c**	**2a**	**3c**	2-thienyl	CO_2_Me	67
**1d**	**2a**	**3d**	2-furyl	CO_2_Me	56
**1a**	**2b**	**4a**	H	COMe	74
**1b**	**2b**	**4b**	phenyl	COMe	53
**1c**	**2b**	**4c**	2-thienyl	COMe	72
**1d**	**2b**	**4d**	2-furyl	COMe	56

^a^ Isolated by column chromatography; ^b^ Reported in reference [16].

**Table 2:** Preparation of 7-aminophenyl-1-arylisoquinolinquinones **5a**–**h** and **6a**–**f**. 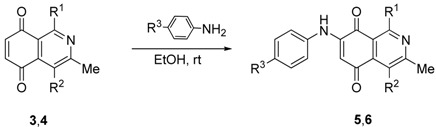

Compound N°	R^1^	R^2^	R^3^	Yield (%) ^a^
**5a**	H	CO_2_Me	H	47 ^b^
**5b**	H	CO_2_Me	OMe	36 ^b^
**5c**	phenyl	CO_2_Me	H	57
**5d**	phenyl	CO_2_Me	OMe	53
**5e**	thien-2-yl	CO_2_Me	H	71
**5f**	thien-2-yl	CO_2_Me	OMe	93
**5g**	fur-2-yl	CO_2_Me	H	65
**5h**	fur-2-yl	CO_2_Me	OMe	76
**6a**	H	COMe	H	98
**6b**	phenyl	COMe	H	70
**6c**	thien-2-yl	COMe	H	77
**6d**	fur-2-yl	COMe	H	91
**6e**	phenyl	COMe	OMe	97
**6f**	thien-2-yl	COMe	OMe	57

^a^ Isolated by column chromatography; ^b^ Reported in reference [17].

### 2.2. Electrochemical Results

The redox potentials of the members of the series were measured by cyclic voltammetry in acetonitrile as solvent, at room temperature, using a platinum electrode and 0.1 M tetraethylammonium tetrafluoroborate as the supporting electrolyte [22]. Well-defined quasi-reversible waves, the cathodic peak related to the reduction of quinone, and the anodic one due to its re-oxidation, were observed for the compounds. The voltammograms were run in the potential range 0–2.0 V *versus* non-aqueous Ag/Ag+. The first half-wave potential values, E^I^_1/2_, evaluated from the voltammograms obtained at a sweep rate of 100 mV s^−1^, are summarized in Table 3.

The E^I^_1/2_ values for the first electron, which are related with the formation of the semiquinone radical anion [23,24], are in the potential range −344 to −588 mV. Analysis of the data in Table 3 indicate that the insertion in **3a** and **4a** of the phenyl, 2-thienyl and 2-furyl groups at 1-position, as in **3b**–**d** and **4b**–**d**, induces the displacement of the half-wave potentials of the former ones (**3a**: −352; **4a**: −344 mV), towards more negative values in the range −384 to −430 mV. Comparison of the E^I^_1/2_ values of compounds **3a**-**d** and **4a**-**d** indicate that the cathodic shifts, attributed to the electron-donating capacity of the aryl groups, are more sensitive to the substitution of the 2-furyl and 2-thienyl groups. We can conclude that the interaction between the aryl-donor and quinone-acceptor molecular fragments makes the reduction of quinones more difficult than that of **3a** and **4a**.

Comparison of the E^I^_1/2_ values of **3a**–**d** and **4a**–**d** respect to their corresponding amination products **5a**–**h** and **6a**–**f** show remarkable cathodic shifts to more negative potentials. This effect is more significant for those compounds containing the 2-thienyl and phenyl substituents at the 1-position. Indeed, the strong electron-donating property of the *p*-anisidino respect to the anilino group produces a major effect of the aforementioned displacement. On the basis of the difference between the E^I^_1/2_ values of **3a**–**d** and **4a**–**d** and their corresponding amination products **5a**–**h** and **6a**–**f** it can be deduced that more favorable donor-acceptor interactions are involved in aminoquinones **5d**, **5f** and **6f**. The differences on the major electron-donating capacity of the *p*-anisidino group respect to the anilino group in compounds **5a**–**h** can also be evidenced by means of the chemical shift of the vinylic proton at C-6. In fact, the vinylic protons of the compounds **5b**, **5d**, **5f**, **5h** containing the *p*-anisidino substituents resonate at higher field (lower δ values) than those of the corresponding anilino-analogues **5a**, **5c**, **5e**, **5g** (higher δ values) (Table 3).

**Table 3:** Half-wave potentials E^I^_1/2_ and proton quinone-chemical shifts of the new compounds. 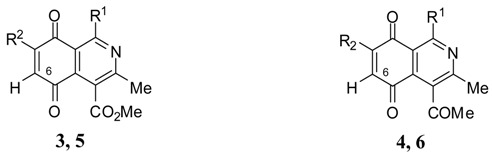

N°	R^1^	R^2^	−E^I^_1/2_ (mV)	6- and/or-7H ^a^
**3a**	H	H	352	7.04
**3b**	phenyl	H	399	6.94 ^b^
**3c**	2-thienyl	H	392	6.99
**3d**	2-furyl	H	415	6.92 ^b^
**4a**	H	H	344	7.05 ^b^
**4b**	phenyl	H	416	6.96 ^b^
**4c**	2-thienyl	H	430	6.99 ^b^
**4d**	2-furyl	H	384	7.00 ^b^
**5a**	H	anilino	563	6.39
**5b**	H	*p*-anisidino	551	6.20
**5c**	phenyl	anilino	560	6.39
**5d**	phenyl	*p*-anisidino	583	6.23
**5e**	2-thienyl	anilino	565	6.39
**5f**	2-thienyl	*p*-anisidino	583	6.18
**5g**	2-furyl	anilino	554	6.38
**5h**	2-furyl	*p*-anisidino	577	6.21
**6a**	H	anilino	464	6.37
**6b**	phenyl	anilino	588	6.39
**6c**	2-thienyl	anilino	576	6.40
**6d**	2-furyl	anilino	533	6.36
**6e**	phenyl	*p*-anisidino	570	6.21
**6f**	2-thienyl	*p*-anisidino	576	6.17

^a^ Recorded in CDCl_3_; ^b^ Average chemical shifts of the 6- and 7-proton signals.

### 2.3. *In Vitro* Antiproliferative Activity of Phenylaminoisoquinolinequinones against Cancer Cell Lines

Aminoisoquinolinequinones **5a**–**h** and **6a**–**f** were evaluated for *in vitro* antiproliferative activity against normal human lung fibroblast MRC-5 and two human cancer cells lines: AGS gastric adenocarcinoma and HL-60 promyelocytic leukemia cells, in 72 h drugs exposure assays. The antiproliferative activity of the new compounds was measured using conventional microculture tetrazolium reduction assays [25,26,27]. The antiproliferative activities by each of the quinones are expressed in terms of IC_50_ (μM) and collected in Table 4. Etoposide, a clinically used anticancer agent, was taken as a positive control.

**Table 4:** Antiproliferative activity of 7-aminophenyl-1-arylisoquinolinequinones **5** and **6**.

N°	IC_50_ ± SEM ^a^ (μM) ^a^			clogP ^e^
	MRC-5 ^b^	AGS ^c^	HL-60 ^d^	
**5a**	2.70 ± 0.60	1.10 ± 0.03	14.81 ± 0.74	0.74
**5b**	2.80 ± 0.80	1.10 ± 0.10	3.80 ± 0.07	0.61
**5c**	5.91 ± 0.36	2.52 ± 0.17	4.39 ± 0.26	2.84
**5d**	>100	>100	>100	2.71
**5e**	9.89 ± 0.51	4.24 ± 0.21	5.19 ± 0.31	2.82
**5f**	9.19 ± 0.53	3.28 ± 0.13	10.26 ± 0.09	2.69
**5g**	4.72 ± 0.29	1.79 ± 0.11	5.0 ± 0.35	1.45
**5h**	4.58 ± 0.35	1.83 ± 0.11	8.04 ± 0.49	1.33
**6a**	3.67 ± 0.22	1.19 ± 0.07	1.24 ± 0.06	0.23
**6b**	5.51 ± 0.22	2.21 ± 0.09	4.74 ± 0.37	2.33
**6c**	>100	>100	>100	2.31
**6d**	5.72 ± 0.24	1.79 ± 0.11	8.19 ± 0.57	0.95
**6e**	16.10 ± 1.11	4.66 ± 0.28	9.36 ± 0.72	2.20
**6f**	>100	4.28 ± 0.21	>100	2.19
**Etoposide**	0.33 ± 0.02	0.58 ± 0.02	2.23 ± 0.09	

^a^ Data represent mean average values for six independent determinations; ^b^ Normal cell line; ^c^ Human gastric adenocarcinoma cell line; ^d^ Promyelocytic leukemia cell line; ^e^ Determined by the ChemBioDraw Ultra 11.0 software.

Comparison of the IC_50_ values obtained with the aminoisoquinolinequinones **5a**–**h** indicates that **5a**, **5b**, **5g** and **5h** are the more potent members against AGS cell line and **5b**, **5c** on the HL-60 cell line. It is worth mentioning that compounds **5a**–**h** have similar half-wave potential values and the more active members (**5a**, **5b**, **5g** and **5h**) exhibited the lower lipophilicity values (Table 4) within this group. Concerning the members of the group **6**, the analogues **6a** and **6d** are the more potent members on the AGS cancer cell line and **6a** and **6b** on the HL-60 cell line. Accordingly, **6a**, having the lowest lipophilicity (clogP = 0.23) and the highest half-wave potential (E^I^_1/2_ = −464 mV) values in the series, emerges as the lead compound. It is noteworthy that even compound **6f** displays a moderate antiproliferative activity on AGS cell line (IC_50_ = 4.28 μM) respect to **6a** (IC_50_ = 1.19 μM); it exhibited the highest selective index (>23) of the series.

In terms of structure-activity relationships the results indicate that the insertion of phenyl, thienyl and furyl substituents at the 1-position of the isoquinolinequinones **5a** and **5b** decreases the antiproliferative activity on AGS cancer cell line compared to the reference compounds. This effect is remarkable when the phenyl group is inserted at the 1-position of **5b**, as in compound **5d**, where the suppression of the antiproliferative activity is observed. Concerning the biological effect of the insertion of the aryl substituents on the HL-60 cancer cell line, an increase was observed of the antiproliferative activity respect to **5a**; however, the insertion in **5b** induces a decreasing effect of the activity. Apparently the antiproliferative activity of compounds **5a**–**h** in the AGS cell line is related in part to hydrophobic factors that are essential for the substances to pass through cell membranes to reach the biological target.

Comparison of the IC_50_ values for compounds **6a**–**f** indicates that the insertion of the aryl group in the 1-position induces a decreasing effect on the antiproliferative activity. This effect is remarkable when the thienyl group is inserted at the 1-position in **6a**, as in compound **6c**, which provoked the suppression of the antiproliferative activity. The antiproliferative activity of compounds **6b**–**f** does not correlate with the E^I^_1/2_ and clogP descriptors.

## 3. Experimental

### 3.1. General

All reagents were commercially available reagent grade and were used without further purification. Melting points were determined on a Stuart Scientific SMP3 apparatus and are uncorrected. ^1^H-NMR spectra were recorded on Bruker AM-400 instrument in deuterochloroform (CDCl_3_). ^13^C-NMR spectra were obtained in CDCl_3_ at 100 MHz. Bidimensional NMR techniques and DEPT were used for signal assignment. Chemical shifts are expressed in ppm downfield relative to tetramethylsilane and the coupling constants (*J*) are reported in Hertz. HRMS data for all final compounds were obtained using a LTQ-Orbitrap mass spectrometer (Thermo-Fisher Scientific, MA 02454, USA) with the analysis performed using an APCI source operated in positive mode. Silica gel Merck 60 (70–230 mesh) was used for preparative column chromatography and TLC aluminum foil 60F_254_ for analytical TLC. Compound **3a** was prepared according to a previously reported procedure [17].

### 3.2. Chemistry

*Methyl 3-methyl-5,8-dioxo-1-phenyl-5,8-dihydroisoquinoline-4-carboxylate* (**3b**)*.* A suspension of (2,5-dihydroxyphenyl)(phenyl)methanone (**1b**, 1 mmol), methyl 3-aminocrotonate (**2a**, 1 mmol), Ag_2_O (2 mmol) and MgSO_4_ (0.5 g) in CH_2_Cl_2_ (25 mL) was stirred at rt for 4 h. The mixture was filtered, the solids were washed with CH_2_Cl_2_ and the solvent removed under reduced pressure. The residue was column chromatographed over silica gel (90:10 CH_2_Cl_2_/EtOAc) to yield pure quinone **3b** (60%) as a yellow solid, mp 175–176.5 °C; IR ν_max_ 1731 (C=O ester), 1678 (C=O quinone); ^1^H-NMR: δ 2.69 (s, 3H, Me), 4.06 (s, 3H, CO_2_Me), 6.98 (d, *J* = 10.3 Hz, 1H, 6-H), 6.90 (d, *J* = 10.3 Hz, 1H, 7-H), 7.00 (m, 5H, phenyl); ^13^C-NMR: δ 23.0, 53.3, 120.9, 125.0, 128.2 (2C), 128.8 (2C), 129.1, 136.8, 139.5, 140.4, 160.1, 160.9, 168.4, 171.1, 183.6, 183.9; HRMS (APCI) calcd. for C_18_H_13_NO_4_: 308.08781 (M + H)^+^; found: 308.09175.

*Methyl 3-methyl-5,8-dioxo-1-(thiophen-2-yl)-5,8-dihydroisoquinoline-4-carboxylate* (**3c**)*.* A suspension of (2,5-dihydroxyphenyl)(thiophen-2-yl)methanone (**1c**, 1 mmol), methyl 3-aminocrotonate (**2a**, 1 mmol), Ag_2_O (2 mmol) and MgSO_4_ (0.5 g) in CH_2_Cl_2_ (25 mL) was stirred at rt for 3.45 h. The mixture was filtered, the solids were washed with CH_2_Cl_2_ and the solvent removed under reduced pressure. The residue was column chromatographed over silica gel (90:10 CH_2_Cl_2_/EtOAc) to yield pure quinone **3c** (67%) as an orange solid, mp 140–142.5 °C; IR ν_max_ 1735 (C=O ester), 1673 (C=O quinone); ^1^H-NMR: δ 2.65 (s, 3H, Me), 4.04 (s, 3H, CO_2_Me), 6.99 (s, 2H, 6- and 7-H), 7.13 (m, 1H, thienyl), 7.54 (m, 1H, thienyl), 7.73 (m, 1H, thienyl); ^13^C-NMR: δ 20.6, 53.1, 119.8, 127.6, 130.4, 131.1, 136.4, 136.8, 140.8, 141.4, 153.0, 159.9, 166.2, 168.3, 183.7, 183.8; HRMS (APCI) calcd. for C_16_H_11_NO_4_S: 314.04423 (M + H)^+^; found: 314.04838.

*Methyl 1-(furan-2-yl)-3-methyl-5,8-dioxo-5,8-dihydroisoquinoline-4-carboxylate* (**3d**)*.* A suspension of (2,5-dihydroxyphenyl)(furan-2-yl)methanone (**1d**, 1 mmol), methyl 3-aminocrotonate (**2a**, 1 mmol), Ag_2_O (2 mmol) and MgSO_4_ (0.5 g) in CH_2_Cl_2_ (25 mL) was stirred at rt for 4.0 h. The mixture was filtered, the solids were washed with CH_2_Cl_2_ and the solvent removed under reduced pressure. The residue was column chromatographed over silica gel (90:10 CH_2_Cl_2_/EtOAc) to yield pure quinone **3d** (56%) as an orange solid, mp 159.5–160.5 °C; IR ν_max_ 1731 (C=O ester), 1665 (C=O quinone); ^1^H-NMR: δ 2.59 (s, 3H, Me), 3.96 (s, 3H, CO_2_Me), 6.52 (m, 1H, furyl), 6.94 (d, *J* = 10.2 Hz, 1- and 6-H), 6.89 (d, *J* = 10.2 Hz, 1- and 7-H), 7.11 (m, 1H, furyl), 7.53 (m, 1H, furyl; ^13^C-NMR: δ 22.8, 53.2, 112.0, 114.3, 120.2, 124.1, 136.4, 136.7, 140.5, 144.8, 148.7, 151.4, 160.1, 168.0, 183.0, 183.6; HRMS (APCI) calcd. for C_16_H_11_NO_5_: 298.06708 (M + H)^+^; found: 298.07113.

*4-Acetyl-3-methylisoquinoline-5,8-dione* (**4a**)*.* A suspension of 2,5-dihydroxybenzaldehyde (**1a**, 1 mmol), 4-aminopent-3-en-2-one (**2b**, 1 mmol), Ag_2_O (2 mmol) and MgSO_4_ (0.5 g) in CH_2_Cl_2_ (25 mL) was stirred at rt for 3.15 h. The mixture was filtered, the solids were washed with CH_2_Cl_2_ and the solvent removed under reduced pressure. The residue was column chromatographed over silica gel (90:10 CH_2_Cl_2_/EtOAc) to yield pure quinone **4a** (74%) as an orange solid, mp 146–148 °C; IR ν_max_ 1706 (C=O ester), 1668 (C=O quinone); ^1^H-NMR: δ 2.58 (s, 3H, Me), 2.62 (s, 3H, COMe), 7.05 (s, 2H, 6- and 7-H), 9.23 (s, 1H, 1-H); ^13^C-NMR: δ 22.7, 30.9, 122.4, 133.3, 133.9, 138.3, 138.9, 148.2, 160.5, 183.6, 184.9, 203.3; HRMS (APCI) calcd. for C_12_H_9_NO_4_: 215.05663 (M + H)^+^; found: 215.05824.

*4-Acetyl-3-methyl-1-phenylisoquinoline-5,8-dione* (**4b**)*.* A suspension of **1b** (1 mmol), 4-aminopent-3-en-2-one (**2b**, 1 mmol), Ag_2_O (2 mmol) and MgSO_4_ (0.5 g) in CH_2_Cl_2_ (25 mL) was stirred at rt for 3.4 h. The mixture was filtered, the solids were washed with CH_2_Cl_2_ and the solvent removed under reduced pressure. The residue was column chromatographed over silica gel (90:10 CH_2_Cl_2_/AcOEt) to yield pure quinone **4b** (53%) as a yellow solid, mp 162.5–164 °C; IR ν_max_ 1707 (C=O ester), 1676 (C=O quinone); ^1^H-NMR: δ 2.61 (s, 3H, Me), 2.64 (s, 3H, COMe), 6.99 (d, *J* = 10.2 Hz, 1- and 6-H), 6.92 (d, *J* = 10.2 Hz, 1- and 7-H), 7.47 (m, 5H, phenyl); ^13^C-NMR: δ 23.0, 31.1, 121.0, 128.4 (2C), 128.8 (2C), 129.2, 133.7, 136.0, 136.7, 139.5, 140.8, 158.7, 160.6, 183.8, 185.1, 203.5; HRMS (APCI) calcd for C_18_H_13_NO_3_: 292.09290 (M + H)^+^; found: 292.09672.

*4-Acetyl-3-methyl-1-(thiophen-2-yl)isoquinoline-5,8-dione* (**4c**)*.* A suspension of **1c** (1 mmol), 4-aminopent-3-en-2-one (**2b**, 1 mmol), Ag_2_O (2 mmol) and MgSO_4_ (0.5 g) in CH_2_Cl_2_ (25 mL) was stirred at rt for 3 h. The mixture was filtered, the solids were washed with CH_2_Cl_2_ and the solvent removed under reduced pressure. The residue was column chromatographed over silica gel (90:10 CH_2_Cl_2_/EtOAc) to yield pure quinone **4c** (72%) as a red solid, mp 161.5–163 °C; IR ν_max_ 1698 (C=O ester), 1671 (C=O quinone); ^1^H-NMR: δ 2.54 (s, 3H, Me), 2.57 (s, 3H, COMe), 7.01 (d, *J* = 10.3 Hz, 1- and 6-H), 6.96 (d, *J* = 10.3 Hz, 1- and 7-H), 7.10 (m, 1H, thienyl), 7.52 (m, 1H, thienyl), 7.73 (m, 1H, thienyl); ^13^C-NMR: δ 22.8, 31.0, 119.7, 127.6, 130.3, 131.0, 133.0, 136.1, 136.6, 141.1, 141.5, 158.1, 170.6, 184.2, 185.1, 202.8; HRMS (APCI) calcd. for C_16_H_11_NO_3_S: 298.04932 (M + H)^+^; found: 298.05313.

*4-Acetyl-1-(furan-2-yl)-3-methylisoquinoline-5,8-dione* (**4d**)*.* A suspension of **1d** (1 mmol), 4-aminopent-3-en-2-one (**2b**, 1 mmol), Ag_2_O (2 mmol) and MgSO_4_ (0.5 g) in CH_2_Cl_2_ (25 mL) was stirred at rt for 3.3 h. The mixture was filtered, the solids were washed with CH_2_Cl_2_ and the solvent removed under reduced pressure. The residue was column chromatographed over silica gel (90:10 CH_2_Cl_2_/AcOEt) to yield pure quinone **4d** (56%) as a yellow solid, mp 159–160 °C; IR ν_max_ 1698 (C=O ester), 1670 (C=O quinone); ^1^H-NMR: δ 2.58 (s, 3H, Me), 2.62 (s, 3H, COMe), 6.61 (m, 1H, furyl), 7.04 (d, *J* = 10.3 Hz, 1- and 6-H), 6.96 (d, *J* = 10.3 Hz, 1- and 7-H), 7.19 (m, 1H, furyl), 7.61 (m, 1H, furyl); ^13^C-NMR: δ 22.8, 31.0, 112.0, 114.2, 120.5, 133.2, 136.0, 136.2, 140.8, 144.8, 148.3, 151.3, 158.6, 183.2, 184.8, 203.3; HRMS (APCI) calcd. for C_16_H_11_NO_4_: 282.07216 (M + H)^+^; found: 282.07584.

### 3.3. General Procedure for the Synthesis of 7-Amino-1-arylisoquinolinquinone Derivatives

A suspension of arylisoquinoline **3**, **4** (1 mmol), the required amine (2 mmol), CeCl_3_x7H_2_O (0.05 mmol) and ethanol (25 mL) was left with stirring at rt after completion of the reaction as indicated by TLC. The solvent was removed under reduced pressure and the residue was column chromatographed over silica gel (90:10 CH_2_Cl_2_/AcOEt) to yield the corresponding 7-amino-1-arylisoquinolinequinone.

*Methyl 3-methyl-5,8-dioxo-1-phenyl-7-(phenylamino)-5,8-dihydroisoquinoline-4-carboxylate* (**5c**)*.* Prepared from **3b** and aniline (7 h, 57% yield): red solid, mp 169.9–171.4 °C; IR ν_max_ 3323 (N-H), 1732 (C=O ester), 1672 (C=O quinone); ^1^H-NMR: δ 2.68 (s, 3H, Me), 4.04 (s, 3H, CO_2_Me), 6.39 (s, 1H, 7-H), 7.19 (m, 3H, arom), 7.39 (m, 2H, arom), 7.45 (m, 5H, phenyl), 7.50 (s, 1H, NH); ^13^C-NMR: δ 23.4, 53.5, 102.9, 120.2, 123.4 (2C), 126.1, 126.6, 128.6 (2C), 128.8 (2C), 129.4, 130.2 (2C), 137.2, 138.7, 140.4, 145.8, 161.1, 161.7, 169.3, 180.7, 181.5; HRMS (APCI) calcd. for C_24_H_18_N_2_O_4_: 399.13001 (M + H)^+^; found: 399.13409.

*Methyl 7-(4-methoxyphenylamino)-3-methyl-5,8-dioxo-1-phenyl-5,8-dihydroisoquinoline-4-carboxylate* (**5d**)*.* Prepared from **3b** and 4-methoxyaniline (5.5 h, 53% yield): red solid, mp 202–203.5 °C; IR ν_max_ 3433 (N-H), 1731 (C=O ester), 1677 (C=O quinone); ^1^H-NMR: δ 2.68 (s, 3H, Me), 3.81 (s, 3H, OMe), 4.04 (s, 3H, CO_2_Me), 6.23 (s, 1H, 7-H), 7.48 (m, 6H, phenyl and NH); ^13^C-NMR: δ 23.5, 53.5, 56.0, 102.2, 115.4 (2C), 120.2, 125.1 (2C), 126.1, 128.6 (2C), 128.8 (2C), 129.4, 129.7, 138.9, 140.4, 145.4, 158.4, 160.8, 161.5, 169.4, 180.8, 181.2; HRMS (APCI) calcd. for C_25_H_20_N_2_O_5_: 429.14058 (M + H)^+^; found: 429.14423.

*Methyl 3-methyl-5,8-dioxo-7-(phenylamino)-1-(thiophen-2-yl)-5,8-dihydroisoquinoline-4-carboxylate* (**5e**)*.* Prepared from **3c** and aniline (3 h, 71% yield): orange solid, mp 163.3–165.3 °C; IR ν_max_ 3428 (N-H), 1699 (C=O ester), 1671 (C=O quinone); ^1^H-NMR: δ 2.65 (s, 3H, Me), 4.02 (s, 3H, CO_2_Me), 6.39 (s, 1H, 7-H), 7.16 (m, 1H, thienyl), 7.24 (m, 3H, arom), 7.43 (m, 2H, arom), 7.56 (m, 1H, thienyl), 7.70 (s, 1H, N-H), 7.73 (1H, thienyl); ^13^C-NMR: δ 23.0, 53.2, 102.5, 119.1, 123.0 (2C), 125.4, 126.5, 127.5, 129.9 (2C), 130.2, 130.7, 136.8, 138.8, 141.6, 145.1, 152.2, 161.1, 168.9, 180.2, 180.9; HRMS (APCI) calcd. for C_22_H_16_N_2_ O_4_S: 405.08643 (M + H)^+^; found: 405.09039.

*Methyl 7-(4-methoxyphenylamino)-3-methyl-5,8-dioxo-1-(thiophen-2-yl)-5,8-dihydroisoquinoline-4-carboxylate* (**5f**)*.* Prepared from **3c** and 4-methoxyaniline (3.15 h, 93% yield): purple solid, mp 214–216 °C; IR ν_max_ 3440 (N-H), 1733 (C=O ester), 1679 (C=O quinone); ^1^H-NMR: δ 2.62 (s, 3H, Me), 3.80 (s, 3H, OMe), 4.00 (s, 3H, CO_2_Me), 6.18 (s, 1H, 7-H), 7.14 (m, 1H, thienyl), 7.54 (m, 1H, thienyl), 7.65 (s, 1H, NH), 7.73 (1H, thienyl); ^13^C-NMR: δ 22.8, 53.0, 56.0, 101.5, 115.0 (2C), 116.5, 119.00, 124.8 (2C), 127.4, 129.4, 130.1, 130.7, 139.2, 141.7, 145.6, 152.8, 158.0, 160.9, 170.0, 180.3, 180.6; HRMS (APCI) calcd. for C_23_H_18_N_2_O_5_S: 435.09700 (M + H)^+^; found: 435.10091.

*Methyl 1-(2-furan-2-yl)-3-methyl-5,8-dioxo-7-(phenylamino)-5,8-dihydroisoquinoline-4-carboxylate* (**5g**)*.* Prepared from **3d** and aniline (3.3 h, 65% yield): red solid, mp 248–249 °C; IR ν_max_ 3441 (N-H), 1774 (C=O ester), 1698 (C=O quinone); ^1^H-NMR: δ 2.66 (s, 3H, Me), 4.02 (s, 3H, CO_2_Me), 6.38 (s, 1H, 7-H), 6.63 (m, 1H, furyl), 7.18 (m, 1H, furyl), 7.24 (m, 3H, arom), 7.42 (m, 2H, arom), 7.63 (m, 1H, furyl), 7.69 (s, 1H, NH); ^13^C-NMR: δ 23.1, 53.2, 102.4, 112.1, 114.2, 119.4, 122.9 (2C), 125.5, 126.4, 129.9 (2C), 137.0, 138.6, 145.0, 145.9, 148.5, 151.8, 161.4, 168.9, 179.8, 181.0; HRMS (APCI) calcd. for C_22_H_16_N_2_O_5_: 389.109288 (M + H)^+^; found: 389.11314.

*Methyl 1-(2-furan-2-yl)-7-(4-methoxyphenylamino)-3-methyl-5,8-dioxo-5,8-dihydroisoquinoline-4-carboxylate* (**5h**)*.* Prepared from **3d** and 4-methoxyaniline (5.3 h, 76% yield): red solid, mp 144–146 °C; IR ν_max_ 3441 (N-H), 1733 (C=O ester), 1611 (C=O quinone); ^1^H-NMR: δ 2.66 (s, 3H, Me), 3.82 (s, 1H, OMe), 4.02 (s, 3H, CO_2_Me), 6.21 (s, 1H, 7-H), 6.63 (m, 1H, furyl), 7.17 (m, 1H, furyl), 7.58 (s, 1H, NH), 7.62 (m, 1H, furyl); ^13^C-NMR: δ 23.1, 53.2, 55.7, 101.7, 112.1, 114.1, 115.1 (2C), 119.6, 125.0 (2C), 125.6, 129.4, 138.8, 144.6, 145.7, 148.5, 151.9, 158.2, 161.3, 169.0, 179.9, 180.7; HRMS (APCI) calcd. for C_23_H_18_N_2_O_6_: 419.11984 (M + H)^+^; found: 419.12360.

*4-Acetyl-3-methyl-7-(phenylamino)isoquinoline-5,8-dione* (**6a**)*.* Prepared from **4a** and aniline (4 h, 98% yield): red solid, mp 178–180 °C; IR ν_max_ 3221 (N-H), 1684 (C=O ester), 1608 (C=O quinone); ^1^H-NMR: δ 2.55 (s, 3H, Me), 2.60 (s, 3H, COMe), 6.37 (s, 1H, 7-H), 7.27 (m, 3H, arom), 7.44 (m, 2H, arom), 7.73 (s, 1H, NH), 9.22 (s, 1H, 1-H); ^13^C-NMR: δ 23.0, 31.2, 103.3, 121.9, 123.3 (2C), 126.6, 130.2 (2C), 134.5, 135.7, 136.7, 145.0, 147.8, 161.7, 180.7, 182.3, 203.7; HRMS (APCI) calcd. for C_18_H_14_N_2_O_3_: 307.10380 (M + H)^+^; found: 307.10776.

*4-Acetyl-3-methyl-1-phenyl-7-(phenylamino)isoquinoline-5,8-dione* (**6b**)*.* Prepared from **4b** and aniline (4.4 h, 70% yield): red solid, mp 181.5–183 °C; IR ν_max_ 3334 (N-H), 1669 (C=O ester), 1655 (C=O quinone); ^1^H-NMR: δ 2.59 (s, 3H, Me), 2.62 (s, 3H, COMe), 6.39 (s, 1H, 7-H), 7.20 (m, 3H, arom), 7.40 (m, 2H, arom), 7.48 (m, 5H, phenyl), 7.64 (s, 1H, NH); ^13^C-NMR: δ 23.0, 31.2, 102.2, 119.9, 122.2 (2C), 126.4, 128.3 (2C), 128.5 (2C), 129.0 (2C), 129.9, 134.1, 136.8, 138.3, 140.0, 145.7, 159.9, 160.4, 180.4, 182.1, 203.6; HRMS (APCI) calcd. for C_24_H_18_N_2_O_3_: 383.13510 (M + H)^+^; found: 383.13899.

*4-Acetyl-3-methyl-7-(phenylamino)-1-(thiophen-2-yl)isoquinoline-5,8-dione* (**6c**)*.* Prepared from **4c** and aniline (5 h, 77% yield): red solid, mp 93.5–94.5 °C; IR ν_max_ 3307 (N-H), 1701 (C=O ester), 1668 (C=O quinone); ^1^H-NMR: δ 2.55 (s, 3H, Me), 2.59 (s, 3H, COMe), 6.37 (s, 1H, 7-H), 7.15 (m, 1H, thienyl), 7.25 (m, 2H, arom), 7.45 (m, 3H, arom), 7.56 (m, 1H, thienyl), 7.76 (m, 1H, thienyl), 7.82 (s, 1H, NH); ^13^C-NMR: δ 22.9, 31.2, 101.6, 119.1, 123.1 (2C), 125.5, 126.5, 127.4, 128.9, 130.0 (2C), 130.6, 133.7, 136.7, 138.8, 146.2, 152.7, 159.8, 180.4, 181.9, 203.3; HRMS (APCI) calcd. for C_22_H_16_N_2_O_3_S: 389.09152 (M + H)^+^; found: 389.09551.

*4-Acetyl-1-(2-furyl)-3-methyl-7-(phenylamino)isoquinoline-5,8-dione* (**6d**)*.* Prepared from **4d** and aniline (4.15 h, 91% yield): red solid, mp 187–188.5 °C; IR ν_max_ 3305 (N-H), 1692 (C=O ester), 1611 (C=O quinone); ^1^H-NMR: δ 2.55 (s, 3H, Me), 2.61 (s, 3H, COMe), 6.36 (s, 1H, 7-H), 6.62 (m, 1H, furyl), 7.11 (m, 1H, furyl), 7.25 (m, 3H, arom), 7.43 (m, 2H, arom), 7.62 (m, 1H, furyl), 7.74 (s, 1H, NH); ^13^C-NMR: δ 23.0, 31.2, 102.1, 112.0, 113.9, 119.6, 123.0 (2C), 126.5, 129.0 (2C),134.0, 136.8, 138.5, 144.6, 146.2, 148.2, 151.9, 160.0, 179.8, 181.5, 203.0; HRMS (APCI) calcd. for C_22_H_16_N_2_O_4_: 373.11436 (M + H)^+^; found: 373.11845.

*4-Acetyl-7-(4-methoxyphenylamino)-3-methyl-1-phenylisoquinoline-5,8-dione* (**6e**)*.* Prepared from **4b** and 4-methoxyaniline (9 h, 97% yield): red solid, mp 162–163 °C; IR ν_max_ 3317 (N-H), 1700 (C=O ester), 1681 (C=O quinone); ^1^H-NMR: δ 2.58 (s, 3H, Me), 2.61 (s, 3H, COMe), 3.80 (s, 3H, OMe), 6.21 (s, 1H, 7-H), 7.48 (m, 5H, phenyl), 7.54 (s, 1H, NH); ^13^C-NMR: δ 23.4, 31.5, 56.0, 101.8, 115.2, 116.8 (2C), 120.3, 128.6 (2C), 128.9 (2C), 129.3, 129.7 (2C), 134.4, 138.9, 140.4, 146.8, 158.5, 160.1, 160.6, 180.8, 182.1, 204.0; HRMS (APCI) calcd. for C_25_H_20_N_2_O_4_: 413.14566 (M + H)^+^; found: 413.14914.

*4-Acetyl-7-(4-methoxyphenylamino)-3-methyl-1-(thiophen-2-yl)isoquinoline-5,8-dione* (**6f**)*.* Prepared from **4c** and 4-methoxyaniline (10 h, 57% yield): red solid, mp 188.5–190 °C; IR ν_max_ 3308 (N-H), 1699 (C=O ester), 1681 (C=O quinone); ^1^H-NMR: δ 2.53 (s, 3H, Me), 2.57 (s, 3H, COMe), 3.81 (s, 3H, OMe), 6.17 (s, 1H, 7-H), 7.15 (m, 1H, thienyl), 7.54 (m, 1H, thienyl), 7.65 (s, 1H, NH), 7.74 (m, 1H, thienyl); ^13^C-NMR: δ 22.9, 31.2, 55.7, 101.3, 115.1 (2C), 119.2, 125.0 (2C), 127.4, 129.4, 130.0, 130.6, 133.8, 139.2, 141.9, 146.9, 152.6, 158.2, 159.5, 180.5, 181.6, 203.3; HRMS (APCI) calcd. for C_23_H_18_N_2_O_4_S: 419.10208 (M + H)^+^; found: 419.10590.

### 3.4. Cell Growth Inhibition Assay

The cell lines used in this work were obtained from the American Type Culture Collection (ATCC, Manasas, VA, USA). They included MRC-5 normal human lung fibroblasts (CCL-171), AGS human gastric adenocarcinoma cells (CRL-1739), and HL-60 promyelocytic leukemia cells (CCL-240). After the arrival of the cells, they were proliferated in the corresponding culture medium as suggested by the ATCC. The cells were stored in medium containing 10% glycerol in liquid nitrogen. The viability of the cells after thawing was higher than 90%, as assessed by trypan blue exclusion test. Cells were sub-cultured once a week and the medium was changed every two days. Cells were grown in the following media: MRC-5 in Eagle minimal essential medium (EMEM), AGS cells in Ham F-12, and HL-60 in suspension in RPM1. The EMEM medium contained 2 mM L-glutamine, 1 mM sodium pyruvate and 1.5 g/L sodium hydrogen carbonate. Ham F-12 was supplemented with 2 mM L-glutamine and 1.5 g/L sodium hydrogen carbonate. RPM1 medium containing 1mM sodium pyruvate and 2.0 g/L sodium bicarbonate. All media were supplemented with 10% heat-inactivated FBS, 100 IU/mL penicillin and 100 μg/mL streptomycin in a humidified incubator with 5% CO_2_ in air at 37 °C. For the experiments, cells were plated at a density of 50,000 cells/mL in 96-well plates. One day after seeding, the cells were treated with the medium containing the compounds at concentrations ranging from 0 up to 100 μM during 3 days. The concentrations used to calculate the IC_50_ values were: 100, 50, 25, 12.5, 6.25, 3.125, 1.56, 0.78, 0.39, 0.195 and 0.00 µM. The compounds were dissolved in DMSO (1% final concentration) and complete medium. Untreated cells (medium containing 1% DMSO) were used as controls. At the end of the incubation, the MTT reduction (3-(4,5-dimethylthiazol-2-yl)-2,5-diphenyltetrazolium bromide) assay was carried out to determine cell viability. The final concentration of MTT was 1 mg/mL. The culture medium containing the compounds under evaluation, was removed from each well by means by vacuum aspiration before adding the MTT solution. MTT metabolite was dissolved adding 100 µL of ethanol (acidified with HCl). The plates were shaken for 10 min and the absorbance was measured at 550 nm using a Universal Microplate Reader (ELx800, Bio-Tek Instruments Inc., Winnoski, VT, USA). Six replicates for each concentration were used and the values were averaged. The results were transformed to percentage of controls and the IC_50_ values were graphically obtained from the dose-response curves. The IC_50_ value was obtained adjusting the dose-response curve to a sigmoidal model (a + (b − a)/1 + 10^(x − c)^), where c = log IC_50_.

## 4. Conclusions

In conclusion, we have described the access for preparing 1-aryl-substituted isoquinolinequinones through a three-step sequence. The half-wave potential values (E^I^_½_) of the members of the series have proven sensitive to the electron-donor effect of the aryl group (phenyl, 2-thienyl, 2-furyl) at the 1-position as well as to the phenylamino groups (anilino, *p*-anisidino) at the 7-position. The results of the screening show that the majority of the members of the series express *in vitro* antiproliferative activity against normal human lung fibroblasts (MRC-5), gastric adenocarcinoma (AGS), and human leukemia cells (HL-60) cell lines. Biological comparative effects as function of the nature of the substituents suggest that lipophilicity is an important factor on the antiproliferative activity. Compounds **5a**, **5b**, **5g**, **5h**, **6a** and **6d** were selected as the most active members of the new series. Compound **6a,** having the lowest lipophilicity (log P = 0.23) and the highest half-wave potential (E^I^_1/2_ = −464 eV) of the series, exhibited the highest antiproliferative activity (IC_50_: 1.19; 1.24 µM) and shows potential for further investigations considering also the selective index values (IS: 3.08; 2.96) higher than those exhibited by etoposide, used as reference drug (IS: 0.57; 0.14).
